# Hyperoxia exposure promotes endothelial–mesenchymal transition and inhibits regulatory T cell function in human pulmonary microvascular endothelial cells

**DOI:** 10.3389/fped.2024.1295868

**Published:** 2024-07-23

**Authors:** Yifan Sun, Chongbing Yan, Yibo Liu, Yating Lin, Bowen Weng, Xiaohui Gong, Cheng Cai

**Affiliations:** Department of Neonatology, Shanghai Children’s Hospital, School of Medicine, Shanghai Jiao Tong University, Shanghai, China

**Keywords:** regulatory T cell, endothelial-mesenchymal transition, human pulmonary microvascular endothelial cell, hyperoxia exposure, bronchopulmonary dysplasia

## Abstract

**Objective:**

This study aims to investigate the effects of hyperoxia exposure on TGF-β1-induced endothelial-mesenchymal transition (EndoMT) and regulatory T cell (Treg)—mediated immunomodulation in human pulmonary microvascular endothelial cells (HPMECs), which could provide a theoretical basis for further studies of the pathogenesis of bronchopulmonary dysplasia (BPD).

**Methods:**

A BPD cell model was established by exposing HPMECs to hyperoxia. Flow cytometry was used to isolate CD4 + CD3 + CD25 + CD127- Tregs from the peripheral blood samples of preterm infants. HPMECs were divided into four groups based on whether they were exposed to hyperoxia and/or co-cultured with Tregs. Quantitative reverse transcription-polymerase chain reaction (qRT-PCR) and enzyme-linked immunosorbent assay (ELISA) were used to test the expression levels of TGF-β1, α-SMA, Foxp3, IL-10, and reactive oxygen species (ROS).

**Results:**

The results showed that the expression levels of TGF-β1 and α-SMA in HPMECs increased at 24 h, 48 h, and 72 h of hyperoxia exposure. In the co-culture group of HPMECs and Tregs, Foxp3 and IL-10 expressions decreased at 48 h and 72 h of hyperoxia exposure. ROS expression increased in the hyperoxia group of HPMECs at 24 h, 48 h, and 72 h of hyperoxia exposure, which were higher than those in the hyperoxia group of HPMECs and Tregs.

**Conclusion:**

These findings suggest that hyperoxia exposure promotes EndoMT in HMPECs and inhibits the immunosuppressive effect of Tregs. Despite this, Tregs still seem could protect HPMECs from oxidative stress injury.

## 1 Introduction

Bronchopulmonary dysplasia (BPD) is a chronic lung disease (CLD) characterized by alveolar simplification and abnormal vascular growth following impaired lung development. It is proven to be associated with morbidity and mortality in preterm infants and significantly impacts the long-term quality of life in survivors (1). Hyperoxia exposure is one of the most important risk factors for BPD (2). Although a large number of studies have been conducted to investigate the pathogenesis of BPD, it is not yet fully understood.

Mesenchymal transition plays an important role in tissue fibrosis. It can promote a significant increase in fibroblasts and a significant decrease in microvascular density, leading to an accelerated fibrosis process (3, 4). TGF-β1 is a major driver of endothelial-to-mesenchymal transition (EndoMT) and epithelial-mesenchymal transition (EMT) and plays a crucial part in chronic obstructive pulmonary disease (COPD) and pulmonary fibrosis (4, 5). It was found that hyperoxia exposure in the BPD rat model induced EMT of alveolar epithelial cells (6, 7). However, the mechanism of EndoMT in the pathogenesis of BPD under hyperoxia exposure is unclear.

Moreover, it has been reported that the numbers of regulatory cells (Tregs) in the cord blood of BPD neonates and non-BPD neonates were significantly different, and the numbers of Tregs in the peripheral blood of BPD neonates changed dynamically during the disease process (8, 9). Treg is a subpopulation of CD4+ T cells with significant immunosuppressive effects that can suppress inflammation and autoimmune responses through the release of anti-inflammatory cytokines such as IL-10 (10). FOXP3 is one of the key transcription factors of Treg and is crucial for the immunosuppressive function of Treg (10, 11). The imbalance between anti-inflammatory and pro-inflammatory immune responses mediated by Tregs and Th17 has been reported to be related to the progression of COPD (10, 12).

Based on these findings, we hypothesized that the effects of hyperoxia exposure on EndoMT and Treg—mediated immunomodulation play important roles in the development of BPD. In this study, the human pulmonary microvascular endothelial cells (HPMECs) model exposed to hyperoxia was established to explore the function of EndoMT and Tregs in BPD, which could provide a theoretical basis for further studies of the molecular mechanism of BPD induced by hyperoxia exposure.

## 2 Methods

### 2.1 Participants and sample collection

The clinical data of preterm infants who were admitted to the Neonatology Department of Shanghai Children’s Hospital in October 2022 was collected. Inclusion criteria: (1) Gestational age (GA) at birth less than 32 weeks; (2) Hospital admission within 7 days after birth. Exclusion criteria: (1) Diagnosis of immunodeficiency disease; (2) Diagnosis of severe congenital malformations. The peripheral blood samples were collected around 7 days after birth for Treg isolation. The study was approved by the ethics committee of Shanghai Children’s Hospital (2022RY003) and informed consent was obtained from all the guardians.

### 2.2 Isolation of Tregs

Peripheral blood lymphocytes were isolated using the MACSprepTMPBMC isolation kit (MiltenyiBiotec, Germany). Anti-human CD3 antibody (317318, BioLegend, USA), anti-human CD4 antibody (300508, BioLegend, USA), anti-human CD25 antibody (302626, BioLegend, USA) and anti-human CD127 antibody (351312, BioLegend, USA) were used. The single-cell suspension was placed in tubes. 1 × 106 cells were taken from each tube to make a blank tube, a CD3 single-staining tube, a CD4 single-staining tube, a CD25 single-staining tube, and a CD127 single-staining tube by adding different antibodies respectively. Meanwhile, 3 × 107 cells were taken and all of the antibodies were added to prepare a whole staining tube. Each tube was centrifuged at 1,500 rpm for 5 min after adding 1 ml PBS. The supernatant was discarded and the 100–200 ul liquid was left for flow staining. In addition to the Blank tube, 1 ul anti-human CD16/32 antibody (130-059-901, Miltenyi, Germany) per tube was used to close the non-specific antigen binding sites on the cell surface. Flow-through antibodies were added based on the instructions. PBS was used to wash off unbound antibodies. CD3 + CD4 + CD25 + CD127- Treg population was sorted by a flow Cytometer (BD Biosciences FACSCalibur, USA). Tregs were then cultured at appropriate density in RPMI medium.

### 2.3 Establish of cell model

HPMECs (ScienCell, USA) were inoculated in six-well plates and cultured to an amount of approximately 8 × 106 cells. The concentration of Tregs was adjusted to 1 × 106/ml. HPMECs were co-cultured with Tregs for two days with a ratio of 10:1. The incubation conditions were 37°C and 5% CO2. The oxygen concentration of the hyperoxia groups was set to 95% and the oxygen concentration of the air groups was set to 21%. HPMECs were divided into four groups (H1, H2, HT1, and HT2) based on whether they were exposed to hyperoxia and/or co-cultured with Tregs (Table 1). The expression of each index was detected at different time points (24 h, 48 h and 72 h) of hyperoxia exposure.

**Table 1:** Groups distribution.

Name	Definitions
H1	HPMECs incubated under conditions of 37°C, 5% CO2 and 21% O2
H2	HPMECs incubated under conditions of 37°C, 5% CO2 and 95% O2
HT1	HPMECs and Tregs co-cultured under conditions of 37°C, 5% CO2 and 21% O2
HT2	HPMECs and Tregs co-cultured under conditions of 37°C, 5% CO2 and 95% O2

### 2.4 Research methods

RNA extraction and quantitative reverse transcription-polymerase chain reaction (qRT-PCR) was performed to detect the expressions of genes on the mRNA level. TRIzol reagent (Invitrogen 15596-026, USA) was used for the extraction of RNA. The RNA quantity and quality were detected by NanoDrop 2,000 Spectrophotometer (Thermo Fisher Scientific, USA). Reverse transcription reactions were performed by referring to the instructions of the Reverse Transcription Kit (TaKaRa RR036B, Japan) to obtain cDNA samples. An ABI 7,500 thermal cycler (Applied Biosystems, Thermo Fisher Scientific, USA) using the following 2-step cycling program was performed for PCR: 95°C for 5 min, 40 cycles at 95°C for 15 s, 60°C for 20 s, and 72°C for 40 s. The PCR primers were provided by KeyGEN BioTECH company (China) (Table 2). GAPDH was used for normalization. The relative expression levels were analyzed by the 2^−ΔΔCt^ method.

**Table 2:** Primers for polymerase chain reaction.

Variables	Sequence (5′–3′)
TGF-β1	F: CACCCGCGTGCTAATGG
	R: ATGCTGTGTGTACTCTGCTTGAACT
α-SMA	F: CGTGGCTACTCCTTCGTG
	R: TGATGACCTGCCCGTCT
Foxp3	F: CAGAGAAGCAGCGGACACTCAATG
	R: AGACTCAGGTTGTGGCGGATGG
IL-10	F: GAGAACCAAGACCCAGACATCAAGG
	R: AAGGCATTCTTCACCTGCTCCAC
GAPDH	F: AGATCATCAGCAATGCCTCCT
	R: TGAGTCCTTCCACGATACCAA

Enzyme-linked immunosorbent assay (ELISA) was performed to detect the expressions of genes on protein level. The TGF-β1 assay kit (mlbio: ml022522, China), α-SMA assay kit (mlbio: ml027532, China), ROS assay kit (KeyGEN BioTECH: KGT010-1, China), Foxp3 ELISA kit (mlbio: ml027353, China) and IL-10 ELISA kit (mlbio: ml064299, China) were used to test the expression of each index respectively. Standard wells, blank wells, and sample wells were set up respectively according to the instructions. 50 ul samples and 50 ul assay reagent were added to each well. The liquid in the wells was discarded after incubation for 30 min at 37°C. The chromogenic agent was added according to the instructions. Finally, optical density (OD) was measured sequentially at 450 nm using a Microplate reader (Rayto RT-6100, USA).

### 2.5 Statistical analysis

The results are presented as the mean ± standard deviation. All experiments were repeated 3 times. *T*-test was used to analyze data with the GraphPad Prism software (GraphPad, USA). *P *< 0.05 was considered statistically significant.

## 3 Results

### 3.1 General clinical information

A total of 14 preterm infants were involved in this study, including 6 male and 8 female infants with a GA of 29.2 ± 1.4 weeks and a Birth weight (BW) of 1,309.3 ± 246.6 g. Five of the 14 preterm infants developed BPD at 36 weeks postmenstrual age.

Peripheral blood samples were collected randomly from 5 of the 14 preterm infants with a GA of 30.2 ± 1.2 weeks and a BW of 1,570.0 ± 177.1 g. The 5 infants included 1 male and 4 females. One of the 5 preterm infants developed BPD at 36 weeks postmenstrual age.

### 3.2 Hyperoxia exposure increases expressions of TGF-β1 and α-SMA

**Figure 1:**
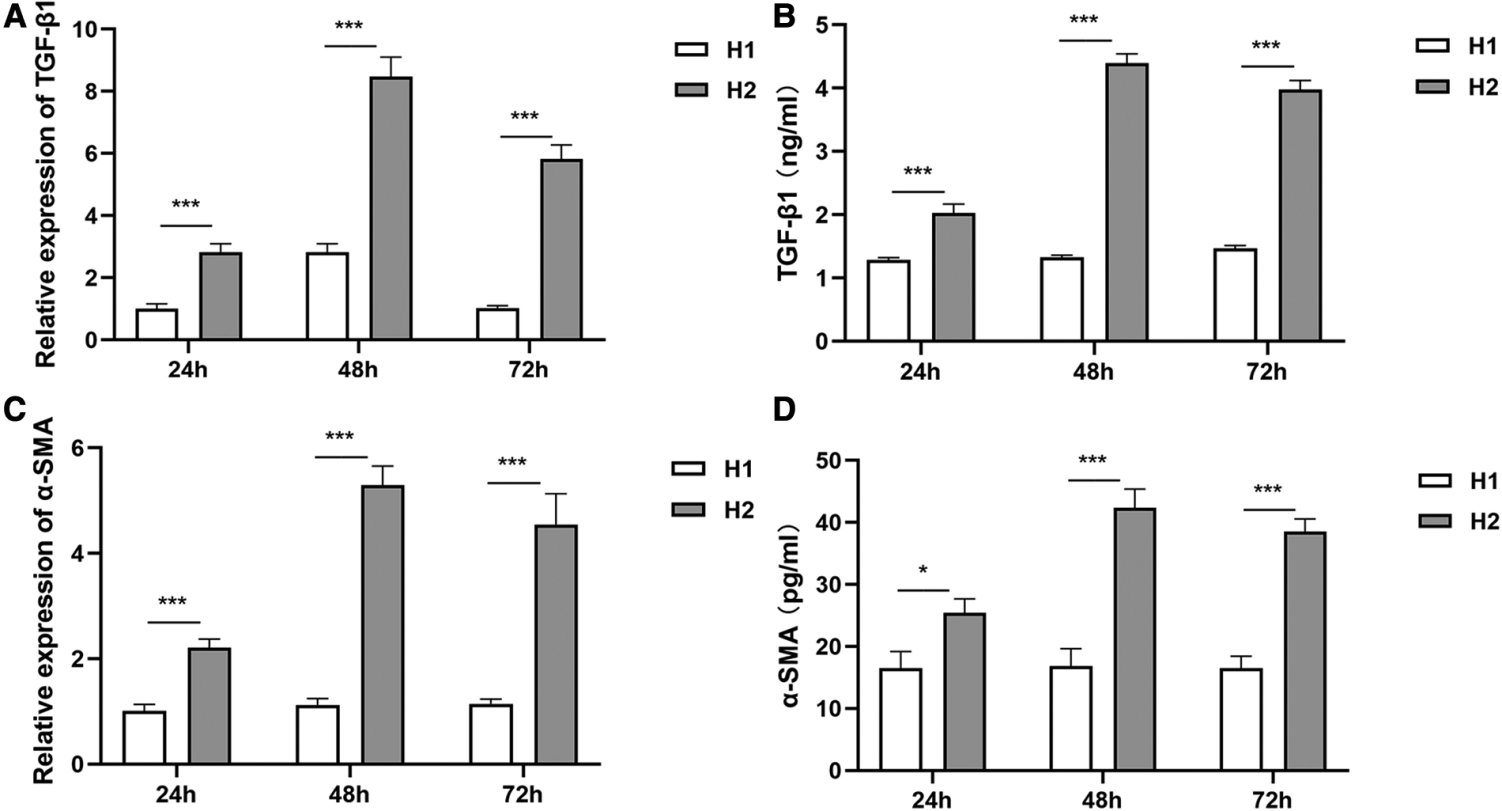
The expressions of TGF-β1 and α-SMA at different time points of hyperoxia exposure. (**A,B**) At 24 h, 48 h, and 72 h of hyperoxia exposure, TGF-β1 expressions at mRNA levels and protein levels in the H2 group (*n* = 3) were significantly higher than those in the H1 group (*n* = 3). (**C,D**) Compared with the H1 group (*n* = 3), α-SMA expressions in the H2 group (*n* = 3) were increased significantly at mRNA levels and protein levels at 24 h, 48 h, and 72 h of hyperoxia exposure. Three independent experimental repeats were performed. *P < 0.05, ***P < 0.001. H1, HPMECs incubated under conditions of 37°C, 5% CO2 and 21% O2; H2, HPMECs incubated under conditions of 37°C, 5% CO2 and 95% O2.

Compared with those in the H1 group, TGF-β1 and α-SMA expressions at mRNA and protein levels in the H2 group increased significantly at 24 h, 48 h, and 72 h of hyperoxia exposure (Figure 1).

### 3.3 Isolation of Tregs from peripheral blood samples

**Figure 2:**
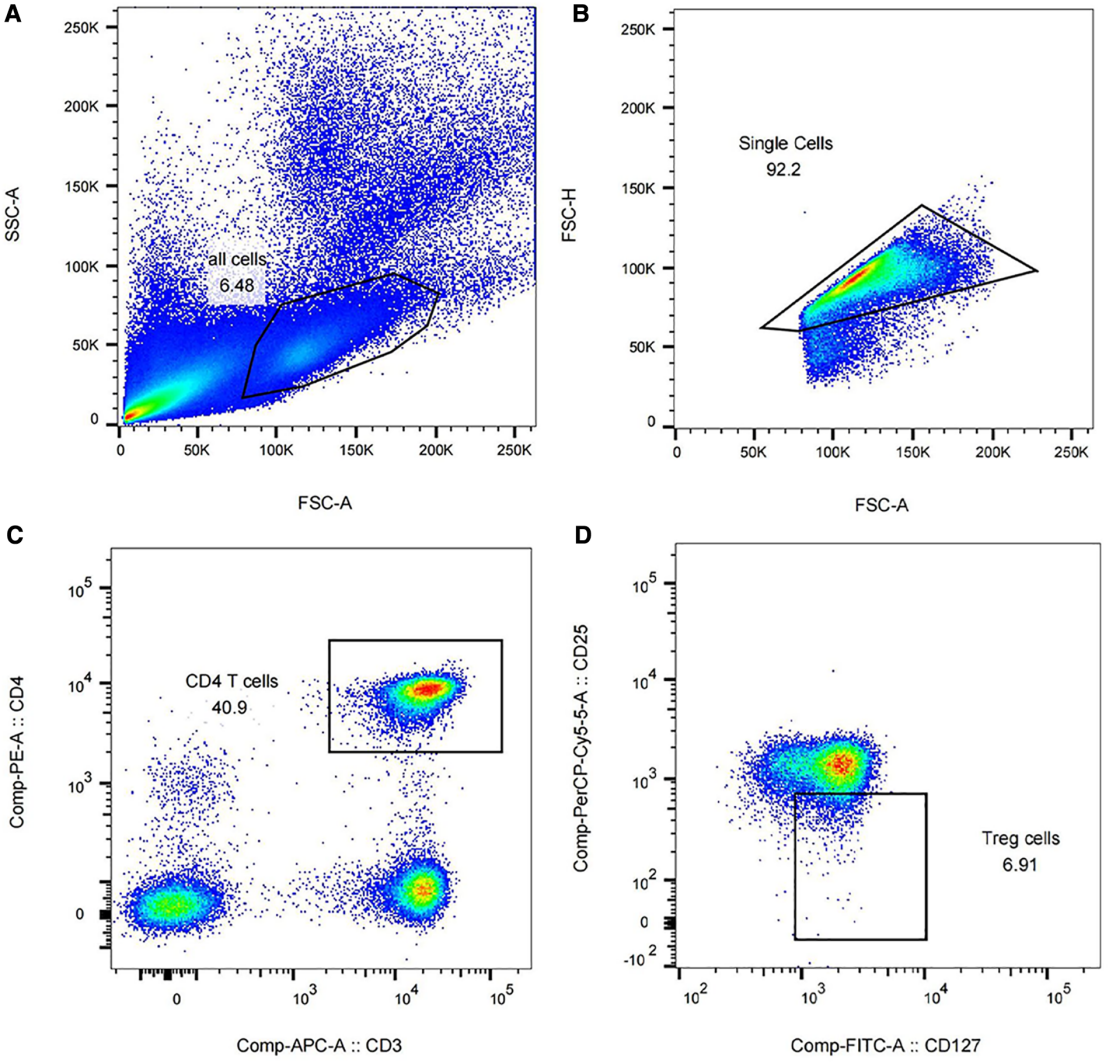
Isolation of Tregs in peripheral blood of preterm infants using flow cytometry (partial results). (**A**) Peripheral blood cells accounted for 6.48% of all peripheral blood components. (**B**) The proportion of monocytes in peripheral blood cells was 92.2%. (**C**) The proportion of CD4 + CD3+ T cells in monocytes was 40.9%. (**D**) CD25 + CD127- Treg accounted for 6.91% of CD4 + CD3+ T cells.

The results of flow cytometry showed that the purity of monocytes in peripheral blood cells reached 90.5%–98.7%. CD4 + CD3+ T cells accounted for 40.9%–53.4% of the isolated monocytes, and CD25 + CD127- Treg accounted for 6.91%–11.9% of the total CD4 + CD3+ T cells isolated. Some of the results are shown in Figure 2.

### 3.4 Hyperoxia exposure decreases expressions of Foxp3 and Il-10

**Figure 3:**
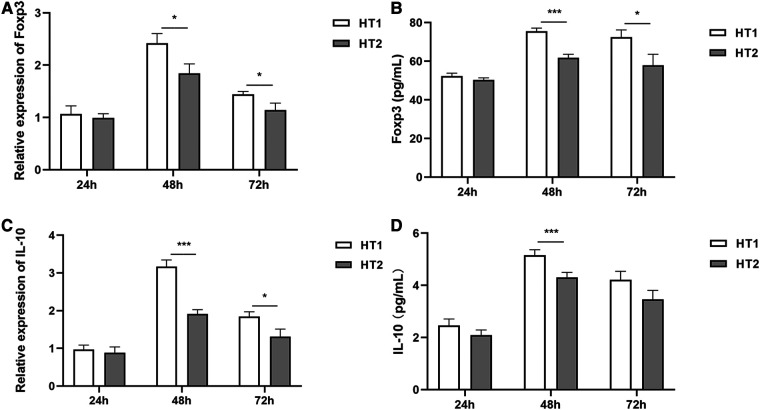
The expression of Foxp3 and IL-10 at different time points of hyperoxia exposure. (**A,B**): Foxp3 expressions showed no difference between the HT1 group (*n* = 3) and HT2 gourp (*n* = 3) at 24 h of hyperoxia exposure. However, at 48 h and 72 h of hyperoxia exposure, Foxp3 expressions decreased significantly in the HT2 group (*n* = 3) compared with those in the HT1 group (*n* = 3). (**C**) Although no significant difference was observed between the HT1 group (*n* = 3) and HT2 group (*n* = 3) after hyperoxia exposure for 24 h, IL-10 expressions at mRNA levels decreased significantly in the HT2 group (*n* = 3) compared with those in the HT1 group (*n* = 3) after hyperoxia exposure for 48 h and 72 h. (**D**) The results of ELISA showed that there was no difference in IL-10 expressions between the HT1 group (*n* = 3) and HT2 group (*n* = 3) at 24 h of hyperoxia exposure. Compared with that in the HT1 group (*n* = 3), a significant decrease was detected in the HT2 group (*n* = 3) at 48 h of hyperoxia exposure. The results also showed that compared with that in the HT1 group, the IL-10 expression in the HT2 group had no significant difference, but a downward trend at 72 h of hyperoxia exposure. Three independent experimental repeats were performed. *P < 0.05, ***P < 0.001. HT1, HPMECs and Tregs co-cultured under conditions of 37°C, 5% CO2 and 21% O2; HT2, HPMECs and Tregs co-cultured under conditions of 37°C, 5% CO2 and 95% O2.

No significant difference in the expressions of Foxp3 and IL-10 was detected between HT1 group and HT2 group at 24 h of hyperoxia exposure (Figure 3). However, at 48 h of hyperoxia exposure, the expressions of Foxp3 and IL-10 were significantly decreased in the HT2 group compared with those in the HT1 group (Figure 3). At 72 h of hyperoxia exposure, expressions of Foxp3 in the HT2 group were lower than those in the HT1 group (Figures 3A–C). Compared with that in HT1 group at 72 h of hyperoxia exposure, IL-10 expression at mRNA level in HT2 group decreased significantly, but its expression at the protein level showed a decreasing trend which is not statistically significant (Figures 3C,D).

### 3.5 Co-culture of HPMEC and Treg inhibits the increase of ROS expression under hyperoxia exposure

**Figure 4:**
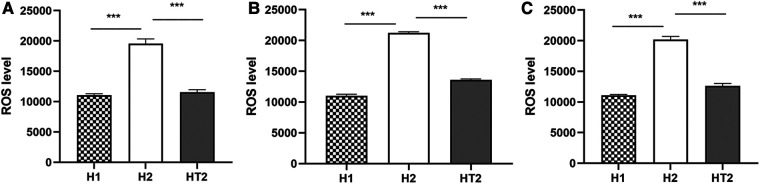
The expression of ROS detected by ELISA at different time points of hyperoxia exposure. (**A–C**) After hyperoxia exposure for 24 h, 48 h, and 72 h, the ROS expressions in the H2 group (*n* = 3) were significantly higher than those in the H1 group (*n* = 3) and HT2 group (*n* = 3). Three independent experimental repeats were performed. ***P < 0.001. H1, HPMECs incubated under conditions of 37°C, 5% CO2 and 21% O2; H2, HPMECs incubated under conditions of 37°C, 5% CO2 and 95% O2; HT2, HPMECs and Tregs co-cultured under conditions of 37°C, 5% CO2 and 95% O2.

The results of ELISA showed that at 24 h, 48 h, and 72 h of hyperoxia exposure, ROS expressions of HPMECs in the H2 group were significantly higher than those in the H1 group (Figure 4). It was also observed that ROS expressions in HT2 group were significantly lower than that in H2 group at 24 h, 48 h and 72 h of hyperoxia exposure (Figure 4).

## 4 Discussion

In this study, we found that the expressions of EndoMT-related indicators (TGF-β1 and α-SMA) were significantly increased in the hyperoxia-exposed HPMEC model. To further explore the interaction between Tregs and HPMECs under hyperoxia exposure, HPMECs were co-cultured with CD4 + CD3 + CD25 + CD127- Tregs which were isolated from the peripheral blood of preterm infants less than 32 weeks of GA. The findings showed that the expressions of Foxp3, a key transcription factor of Treg, and IL-10, an anti-inflammatory cytokine secreted by Treg, were both decreased after hyperoxia exposure, but Tregs still had a significant immunosuppressive effect on the increase of ROS expression induced by hyperoxia exposure.

The pathological feature of the new “BPD” is the retardation of lung development leading to simplification of alveolar structure and abnormities of pulmonary vascular development (1). HPMEC is crucial for the induction of pulmonary microangiogenesis and has important physiological functions such as constituting the endothelial barrier and secretion (13, 14). Pulmonary microvasculature actively promotes alveolar growth during lung development and helps maintain normal alveolar structure (13, 14). Our study found that TGF-β1 and α-SMA expressions increased significantly in the hyperoxia-exposed HPMEC cell model, confirming that hyperoxia exposure induces EndoMT in HPMECs. EndoMT can be triggered by different mechanisms in different cell types and cellular physiological or pathological states and involves different signaling pathways (15). EndoMT is also vital for embryonic development, malignancy, angiogenesis, inflammation, and fibrotic diseases (15). It has been reported that the EndoMT in HPMECs mediated by the TGF-β1/Smad3 pathway, and the EMT in lung epithelial cells can contribute to the development of COPD and pulmonary fibrosis (4, 5). It has also been found that EndoMT promotes pulmonary vascular remodeling in pulmonary arterial hypertension (15, 16). Therefore, EndoMT in HPMECs induced by hyperoxia exposure may contribute to the development of BPD.

T-cell counts and function have been reported to be involved in the development of BPD-associated lung injury (17–19). In our study, Tregs co-cultured with HPMECs were isolated from the peripheral blood of preterm infants with GA less than 32 weeks. A significant decrease in Foxp3 and IL-10 expressions under hyperoxia exposure was found, suggesting that hyperoxia exposure may inhibit the immunomodulatory function of Tregs. Treg is an important subpopulation of Th cells and is central to the maintenance of immune homeostasis (20). Foxp3 is a nuclear transcription factor of Treg, which is essential for Treg to exert immunosuppressive function (21, 22). Treg secretes immunosuppressive factors such as IL-10 and TGF-β, which are involved in a variety of chronic inflammation-mediated lung diseases (9, 23). Treg has the potential to shift from the anti-inflammatory phenotype to the pro-inflammatory phenotype under specific conditions (24). Treg of preterm infants exhibits distinct phenotypes and quantitative dynamics in the early postnatal period compared with that of term infants and adults (9). Lee and his collogues found that an imbalance between pro-inflammatory and anti-inflammatory immune responses due to a decrease in Treg numbers is associated with the development of COPD (25). Misra et al. collected cord blood samples from newborns at birth and found that Treg expression was significantly higher in the cord blood of preterm infants compared with that of term infants. They also found that the absolute number of Tregs in the cord blood of neonates with moderate BPD was significantly lower than that of patients with no or mild BPD (8). The results of Pagel et al. demonstrated that the number and immunosuppressive function of Tregs were significantly higher in preterm infants compared with that in term infants and adults. Their findings also showed that preterm infants with BPD had a significant increase in Treg frequency in the early postnatal period, with a peak at 4–10 days postnatal (9). The above results suggest that Treg plays an important role in the pathogenesis of BPD in preterm infants, but the dynamics and molecular mechanisms of Tregs in BPD are not yet clear, and more studies are needed.

In addition, hyperoxia exposure can lead to the release of ROS, causing severe lung injury (2, 23, 26). Previous studies have found that hyperoxia exposure induces oxidative stress in HPMECs, leading to increased cell edema, dysfunction, impaired survival, and abnormal growth (27, 28). Preterm infants are poorly tolerant to hyperoxia and more susceptible to ROS-mediated oxidative stress damage, which can cause lung injury and developmental impairment (29). Our findings demonstrated that hyperoxia exposure caused a significant increase in ROS expression in HPMECs, which was significantly decreased when HPMECs were co-cultured with Tregs. These findings indicated that Tregs had a protective effect on HPMECs under hyperoxia exposure.

In conclusion, these results indicate that hyperoxia exposure promotes the development of EndoMT in HPMEC and attenuates the immunosuppressive effect of Tregs, but Tregs still seem to have a protective effect against oxidative stress injury in HPMECs. Therefore, EndoMT and Tregs may play important roles in hyperoxia-induced lung injury in preterm infants with BPD. However, the molecular mechanism still needs further exploration.

## Data Availability

The original contributions presented in the study are included in the article/Supplementary Material, further inquiries can be directed to the corresponding author.
